# Simultaneous subacute interstitial nephritis and anticoagulant-related nephropathy related to novel oral anticoagulants use

**DOI:** 10.1080/0886022X.2021.2014338

**Published:** 2022-01-27

**Authors:** Zhen-Ling Deng, Wen-Ling Yang, Xin-Yue Zhao, Zi-Yong Tang, Dan-Xia Zheng, Yue Wang

**Affiliations:** Department of Nephrology, Peking University Third Hospital, Beijing, China

**Keywords:** Anticoagulants-related nephropathy, dabigatran, acute kidney injury, subacute interstitial nephritis, hemoglobin immunohistochemical staining

## Abstract

**Introduction:** Interstitial nephritis related to novel oral anticoagulants was only reported in sporadic case reports and none was accompanied by anticoagulants related nephropathy (ARN).

**Case Report:** We presented here a case of biopsy-proven subacute interstitial nephritis (SubAIN) accompanied by ARN after oral dabigatran to alarm clinicians. This case manifested with gross hematuria, acute kidney injury, slightly prolonged thrombin time, moderate anemia, moderate proteinuria, a large quantity of intratubular hemoglobin casts confirmed by hemoglobin antibody immunohistochemical staining which presumed to occur around 1 week after dabigatran and subacute interstitial nephritis accompanied by focal proliferative glomerulonephritis. Serum creatinine level did not continue to elevate after discontinuation of the oral anticoagulant. With the subsequent supportive therapy, it decreased to some extent then reduced to normal with the help of prednisone (half of the full dose).

**Conclusions:** When we came across a patient who manifested as hematuria or acute kidney injury with a history of anticoagulants usage, we should think of ARN and pay more attention on history collection. Secondly, subacute interstitial nephritis may coexist with ARN. Thirdly, hemoglobin immunohistochemical staining may be helpful to make it clear whether the intra-tubular protein casts came from red blood cells. In addition, for those patients who may have decreased kidney function, anticoagulants dose should be reduced to prevent the occurrence of ARN.

Dear Editor,

Anticoagulants-related nephropathy (ARN) came into the public view since the novel oral anticoagulants (NOAC) entered the medical insurance directory. Similarly, warfarin is also known to have adverse renal affects by causing microhemorrhage. However, there is some uncertainty and obstacles in the diagnosis, we are not familiar with ARN. The typical case manifests as unexplained acute kidney injury with diffuse glomerular hemorrhage and often acute tubular necrosis due to renal tubular obstruction by red blood cell casts, accompanied by gross hematuria or not [1,2]. Interstitial nephritis related to NOAC was only reported in sporadic case reports and none was accompanied by ARN [3–7]. Recently we came across a case of biopsy-proven subacute interstitial nephritis accompanied by ARN after oral dabigatran. We would like to present it here for alarm of similar cases. The patient had given the written informed consent to publish her case.

A 62 years old woman with hypertension and paroxysmal atrial fibrillation was admitted because of gross hematuria and elevation of serum creatinine. One month ago, she was prescribed dabigatran (110 mg twice a day) for paroxysmal atrial fibrillation and a high Congestive Heart Failure, Hypertension, Age, Diabetes, Stroke/Transient Ischemic Attack (CHADS_2_) score of 1, the simplified Canadian Cardiovascular Society Algorithm for patients of non- valvular atrial fibrillation. Gross hematuria occurred a week later with frequent urination at night (4-5 times a night) without pain or urgency when she passed urine. A week ago, her serum creatinine was elevated (215.0 μmol/L) with moderate anemia (hemoglobin 80 g/L). Urinalysis demonstrated massive normal (100%) red blood cells and moderate proteinuria (2426.0 mg/(g.cr)). Phospholipase A_2_ receptor (PLA_2_R) antibody was positive with the titer of 33.567 RU/mL (normal value: <20 RU/mL). She was advised to withdraw the dabigatran and valsartan. When she was admitted, she had no more gross hematuria.

Tests in the ward demonstrated moderate anemia with hemoglobin of 76 g/L with normal transferrin saturation (24.0%), ferritin (75.6 ng/mL) and folic acid. Antinuclear antibodies, anti-neutrophil cytoplasmic antibodies, anti-glomerular basement membranous antibodies were all negative. Phase-microscope of urine demonstrated normal 45%, ring-like 15% and crumpled red blood cells 5%. The urinary osmotic pressure was 367.0 mOsm/(kg.H2O) with normal blood osmotic pressure. Enhanced echo in the kidney parenchyma and no obstruction of urinary tract were demonstrated by the ultrasound. Magnetic resonance urography, computed tomography scan of urinary system and cystoscopy found nothing remarkable.

The coagulation test demonstrated prolonged thrombin time (24.2 s, normal value: 12–18) and increased thrombin time ratio (1.67, normal value: 0.8–1.2), which turned normal five days later. Percutaneous ultrasound-guided kidney biopsy was performed which demonstrated subacute tubular interstitial lesion accompanied by focal proliferative glomerulonephritis (Figure 1). Immunofluorescence staining was negative. However, there was no evidence of membranous nephropathy by either electron microscopy or light microscopy.

**Figure 1.**
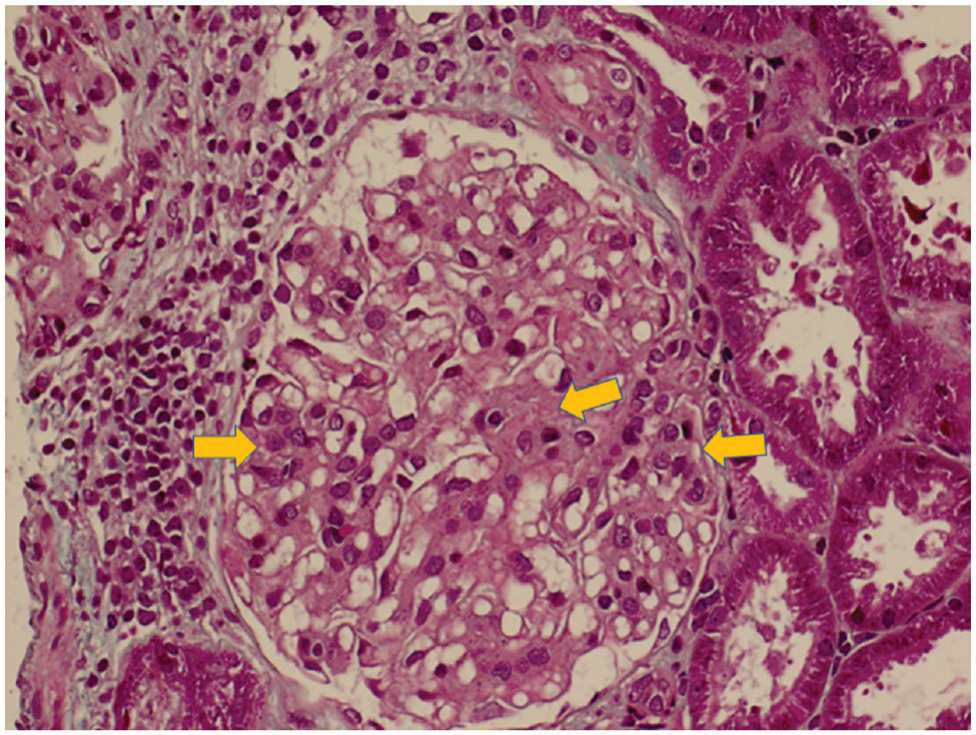
Focal proliferative glomerulonephritis accompanied by mononuclear cells infiltration in the interstitium – hematoxylin and eosin staining (200×). Arrowheads indicated the proliferation of mesangial cells, while the middle arrowhead also showed the increased mesangial matrix.

Interesting finding was a lot of intra-tubular protein casts (Figure 2). We further examined the specimens with human hemoglobin antibody staining (Abcam Company Ltd, item number: ab92492) according to instructions of the manual and confirmed the protein in the tubules to be hemoglobin (Figure 3), which was fit for ARN.

**Figure 2.**
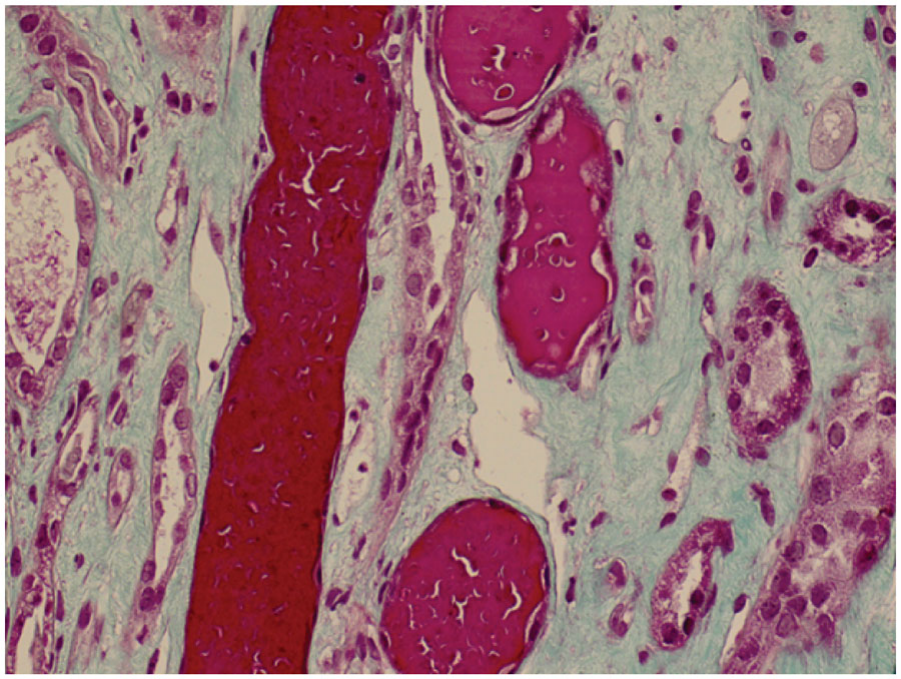
Protein cast in light microscopy – Masson staining (200×).

**Figure 3.**
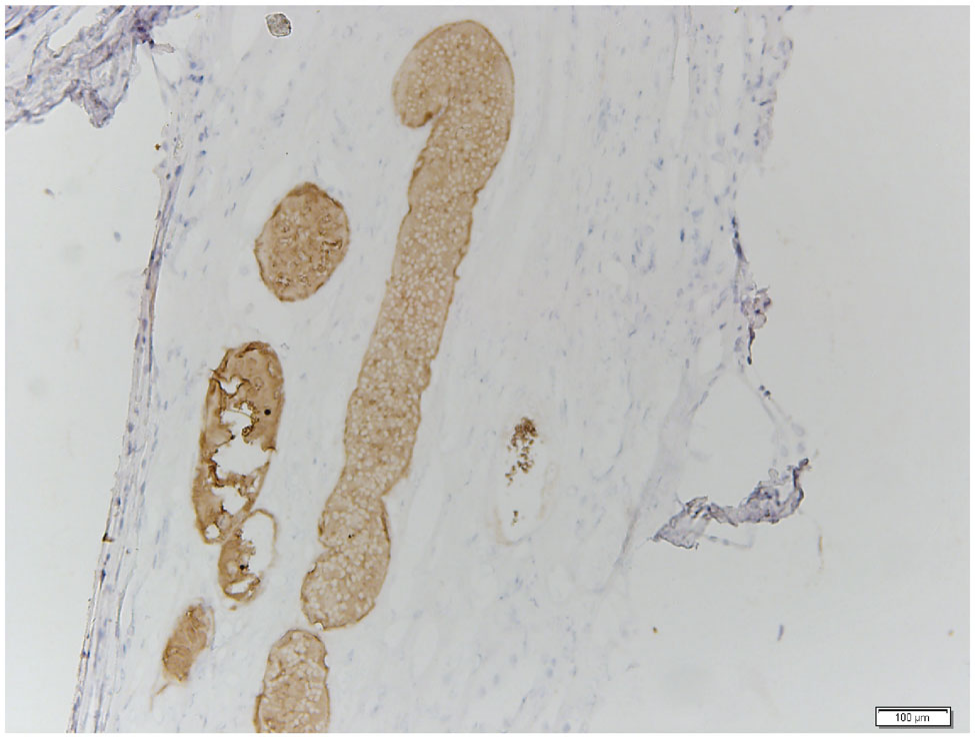
Hemoglobin immunohistochemical staining results in the renal medulla (200×).

Eleven days after kidney biopsy, prednisone 25 mg/day was prescribed since serum creatinine did not return normal after supportive therapy. Three weeks later, her serum creatinine level reduced to 103 μmol/L with a serum hemoglobin of 109 g/L. Serum creatinine level turned normal at 53 days (89 μmol/L) after steroid and remained normal during the follow-up of more than 1 year with minor proteinuria (0.2–0.3 g/d) and no anemia (hemoglobin 118–120 g/L, Supplementary Table 1). Prednisone dose was gradually reduced and stopped after 3 months under the supervision of physicians. Thereafter, aspirin replaced anticoagulants.

**Table 1.** Comparison of literatures of interstitial nephritis as the cause of novel oral anticoagulants related acute kidney injury (AKI).

Author	NOAC	Kidney biopsy	AKI pathology	Age, years	Sex	Preliminary CKD	Diabetes mellitus	Other complications	NOAC dose, mg/d	NOAC period, months
Abdulhadi [7]	Apixaban	0	AIN	76	F	1, CKD 4	1	Pulmonary hypertension	5	6
Patel [4]	Dabigatran	1	AIN + CIN, nodular DN^a^	59	M	1, CKD 3	1	Osteomyelitis	NA	1
DiMaria [6]	Apixaban	1	AIN and mild IgAN	70	M	0	0	Hyperlipidemia	10	12
Zafar [5]	Rivaroxaban	0	AIN	76	M	1, CKD 3	0	Past pulmonary embolism; DVT	NA	1 week
Monahan [3]	Rivaroxaban	1	AIN	82	M	1, CKD 3b	0	A pacemaker for a 3^0^AVB	15	0.5
Marcelino [9]	Rivaroxaban	0	AIN	82	M	NA	1	Dyslipidemia, hyperuricemia	20	2 weeks
This case	Dabigatran	1	SubAIN, intratubular hemoglobin casts	62	F	0	0	None	220	3 weeks

Author	Baseline serum creatinine value, μmol/L	Peak serum creatinine value, μmol/L	Gross hematuria or not	Other bleeding	Proteinuria, g/day	Fever	Rash	Eosinophiluria	Initial pred. dose, mg/day	Period on steroids, months	Scr values after steroids
Abdulhadi [7]	248	1282	0	0	0	0	0	1	1 mg/kg	0.5	433.04 μmol/L
Patel [4]	115	448	0	Gastrointestinal bleeding	NA	0	0	1	NA	NA	2.73 mg/dL at 1 week
DiMaria [6]	74	339	1	0	0.57 g/gcr	0	0	NA	60	2	1.3 mg/dL over a 4-month period
Zafar [5]	221	743	1	0	NA	0	1	1	Methyl-pred.250 mg IV *4 days, then pred. 60	A few weeks.	About 3.3 mg/dL on the 7th day
Monahan [3]	eGFR 39 ml/ (min.1.73 m^2^)	573 (eGFR 8 ml/ (min.1.73 m^2^))	0	0	0.3	0	0	0	40	Unknown	eGFR 34 ml/(min.1.73 m^2^).
Marcelino [8]	NA	215	0	Asymptomatic subdural hematomas after falls	0.72	1	1	0	/	/	86 μmol/L after stopping rivaroxaban
This case	84	215	1	Transient epistaxis	1.5	0	0	0	25	3	Less than 90 µmol/L over a period of more than 1-year.

CKD: chronic kidney disease; DVT: deep vein thrombosis in the right lower extremity; AVB: atrioventricular block; IgAN: IgA nephropathy; NA: not applicable; steroids, Glucosteroids; Pred.: prednisone.

^a^Diabetic glomerulosclerosis.

We presented here a case of biopsy-proven subacute interstitial nephritis accompanied by intra-tubular hemoglobin casts, one of the characteristics of ARN, caused probably by dabigatran with a long follow-up of more than a year, which has not been published in literatures. This case manifested with gross hematuria, acute kidney injury, slightly prolonged thrombin time, moderate anemia and a large quantity of intra-tubular hemoglobin casts which presumed to occur around 1 week after dabigatran. Serum creatinine level elevated by more than 50% with the peak value of 215 μmol/L then it did not continue to elevate after discontinuation of dabigatran. With the subsequent supportive therapy, it decreased to 123 μmol/L then reduced to 89 μmol/L with the help of prednisone (half of the full dose). The patient had no past kidney diseases but kidney biopsy demonstrated focal proliferative glomerulonephritis and minor microscopic hematuria could be traced back to 43 days before admission. In the meanwhile, subacute interstitial nephritis rather than acute tubular necrosis may be one of the causes of acute kidney injury. To our knowledge, ours is the first report of diffuse intra-tubular hemorrhage confirmed by hemoglobin antibody immunohistochemical staining and the first report of simultaneous interstitial nephritis and some characteristics of ARN probably caused by dabigatran in a senior patient [1,3–7] (Table 1).

The pathophysiological mechanism behind NOAC-induced AIN is not known, which was presumed to be associated with either a type I or type IV [5] or cell-mediated hypersensitivity reaction [6]. Besides, the obstruction of hemoglobin cast, blood loss and metabolites of bleeding may injure the tubular epithelial cells [8]. Even though antibody against PLA_2_R has high specificity for idiopathic membranous nephropathy, it can occur in other etiologies such as AIN and diabetic nephropathy [9]. Unfortunately, the biopsy specimen was not tested for PLA_2_R antigens.

Obviously, the protein casts rather than red blood cell casts in this case were remarkable. Even though the hemosiderin could be detected by Prussian blue staining, it may be negative in the acute-phase of hemorrhage when hemoglobin has not degraded, the hemoglobin immunohistochemical staining filled the gap.

Several key points may be derived from this case. At first, when we came across a patient who manifested as hematuria or acute kidney injury with a history of anticoagulants usage, we should think of ARN and pay more attention on history collection. Second, subacute interstitial nephritis may coexist with ARN. Third, hemoglobin immunohistochemical staining may be helpful to make it clear whether the protein casts came from red blood cells. In addition, for those patients who may have decreased kidney function, anticoagulants dose should be reduced to prevent the occurrence of ARN.

## Supplementary Material

Supplementay Figure 3

Supplementary Figure 2

Supplementary Figure 1

Supplementary Table
