# Supplementary figures and images for: Simultaneous subacute interstitial nephritis and anticoagulant-related nephropathy related to novel oral anticoagulants use
Source: Ren Fail. 2022 Jan 27;44(1):30–3. doi: 10.1080/0886022X.2021.2014338 (PMC8803099; doi:10.1080/0886022X.2021.2014338)

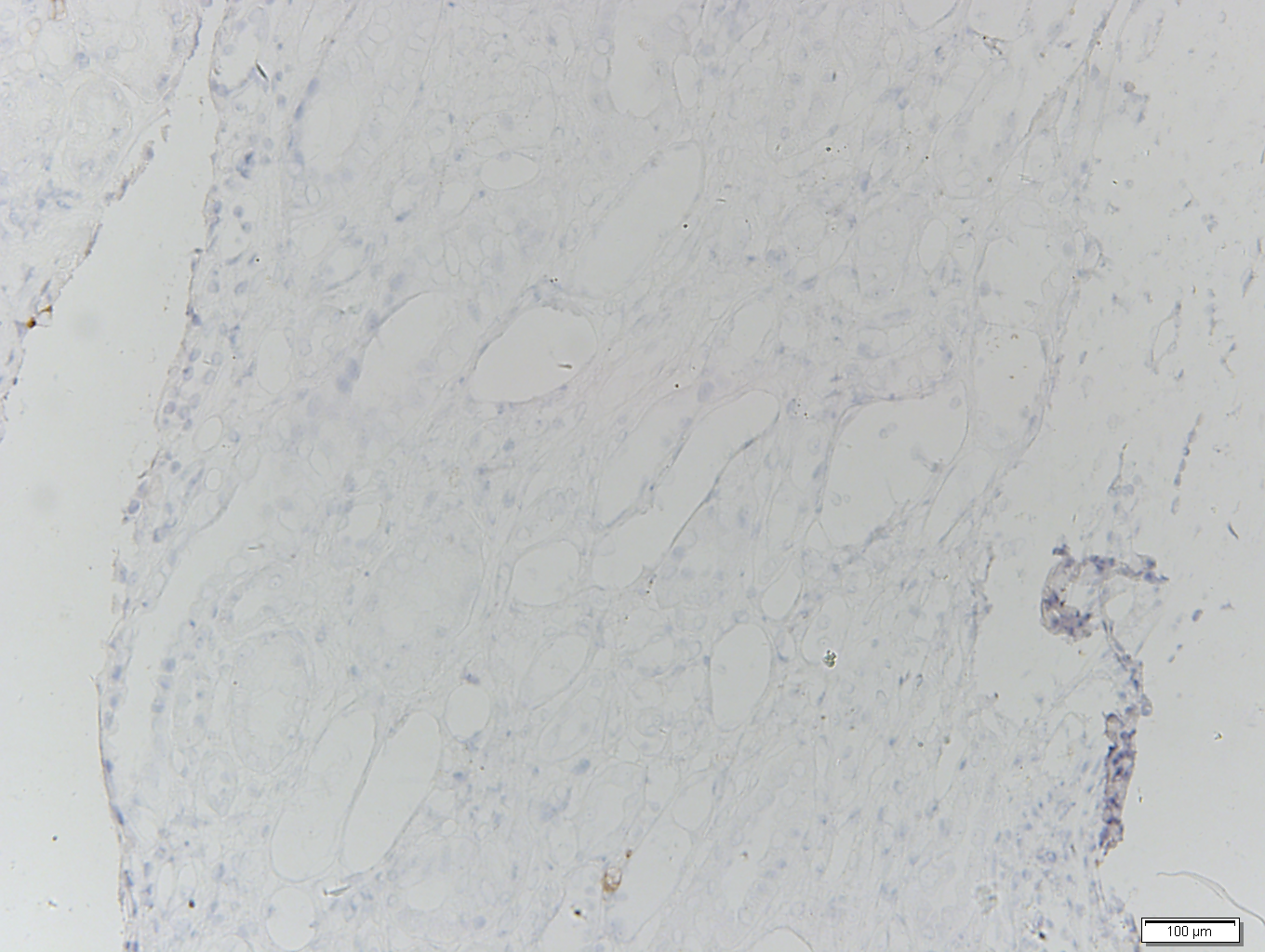

Supplement: Supplementay Figure 3 [file IRNF_A_2014338_SM1619.tif]

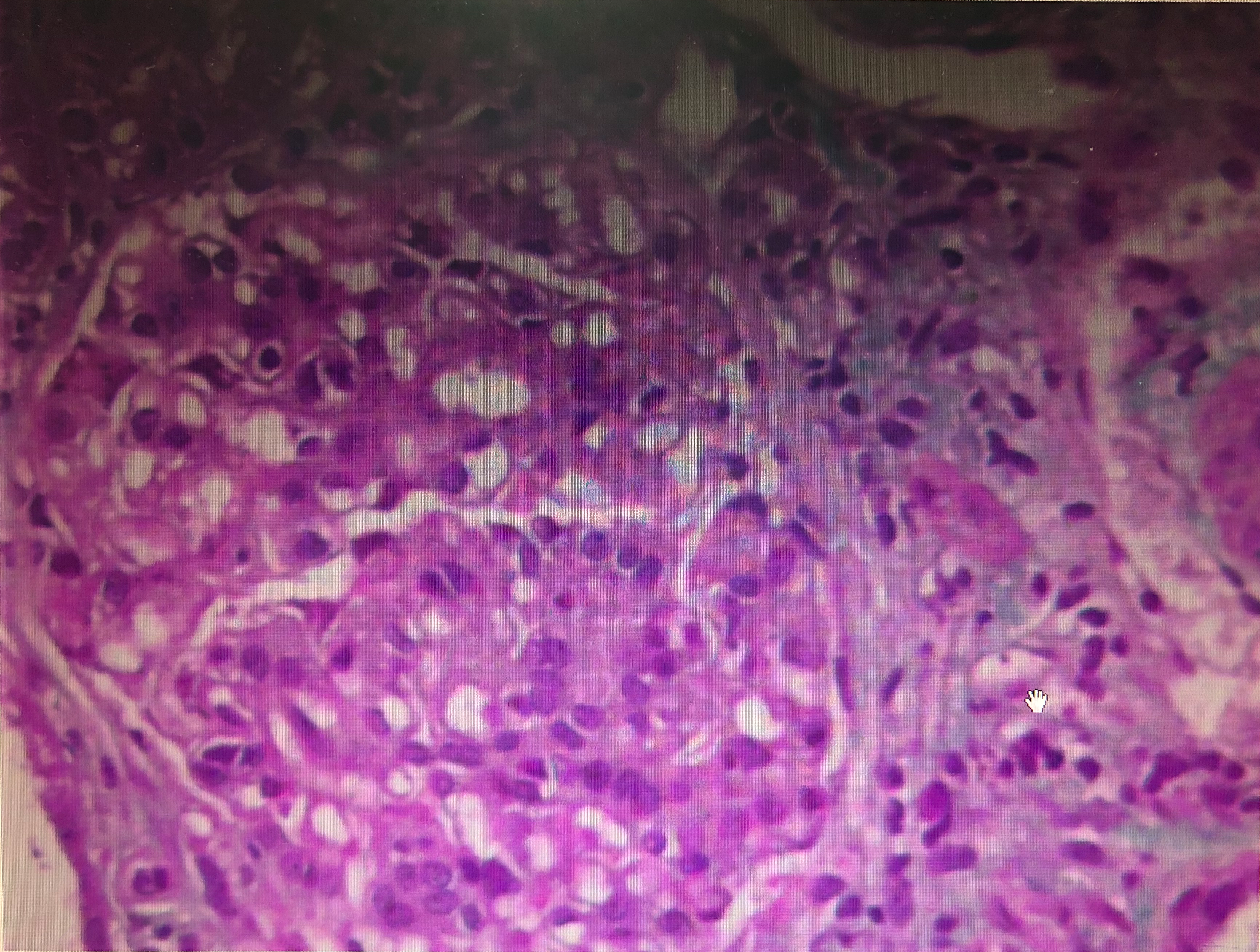

Supplement: Supplementary Figure 2 [file IRNF_A_2014338_SM1390.tif]

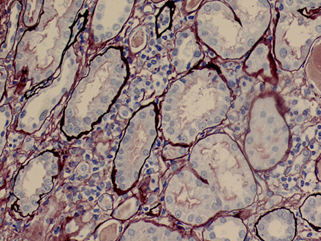

Supplement: Supplementary Figure 1 [file IRNF_A_2014338_SM1354.tif]
