# Supplementary material for: Simultaneous subacute interstitial nephritis and anticoagulant-related nephropathy related to novel oral anticoagulants use
Source: Ren Fail. 2022 Jan 27;44(1):30–3. doi: 10.1080/0886022X.2021.2014338 (PMC8803099; doi:10.1080/0886022X.2021.2014338)
Supplement: Supplementary Table [file IRNF_A_2014338_SM1239.pdf]

Supplementary Table 1: Laboratory results of the reported case.

Time point, days	-56	-43[a]	-6[b]	-1	0, Admitted	2	4	7	11	18[c]	36	71	133	201	469
**HB, g/L**	**117**	**109**	**80**	**82**	**76**	**66**	**71**	**75**	**72**	**73**	**109**	**120**	**120**	**118**	
Bun, mmol/L	7.1	9.2	12.4	12.5	12.3	12	11.4	8	6.8	8.8	8.8	5.7	4.9	5.5	6.4
**Scr, umol/L**	**84**	**89.9**	**215**	**195**	**200**	**179**	**161**	**143**	**123**	**116**	**103**	**89**	**83**	**79**	**73**
uRBC, /HP	[d]	2-4	Full[e]	Full[e]	Full[e]	Full[e]	Full[e]	Full[e]	Full[e]	1-2	0-2	20-22	6-10	8-10	
uRBC, /uL	[d]		5489	343	988		1316	1210	1518	726	1	221	102	97	
PCR, mg/gCr	[d]		2426		1020		1552	1152	1248						668
**UPro, mg/d**	[d]				**1546**			**1002**	**1810**		**373**	**284**	**336**	**227**	/

Note: HB, hemoglobulin; Bun, blood urea nitrogen; Scr, serum creatinine; uRBC, Urinary red blood cell count; PCR, protein/creatinine ratio; ACR, Albumin/creatinine ratio; UPro, Urinary proteinuria

[a] Health checkup. Dabigatran was prescribed at 35 days prior to admission.

[b] Dabigatran withdrawal

[c] Prednisone was added 25 mg per day at 16 days after admission.

[d] no urine test in the health checkup. [e] Full field
